# A Cross-Sectional Study on the Biomechanical Effects of Squat Depth and Movement Speed on Dynamic Postural Stability in Tai Chi

**DOI:** 10.3390/life15060977

**Published:** 2025-06-18

**Authors:** Wenlong Li, Minjun Liang, Liangliang Xiang, Zsolt Radak, Yaodong Gu

**Affiliations:** 1 Faculty of Sports Science, Ningbo University, Ningbo 315211, China; liwenlong21000@163.com (W.L.); guyaodong@nbu.edu.cn (Y.G.); 2 KTH MoveAbility Lab, Department of Engineering Mechanics, KTH Royal Institute of Technology, 114 28 Stockholm, Sweden; 3 Research Institute of Sport Science, Hungarian University of Sport Science, 1123 Budapest, Hungary; radak.zsolt@tf.hu

**Keywords:** traditional Chinese exercises, sports biomechanics, squat movement, postural stability, center of pressure

## Abstract

This study aimed to explore the independent and interactive effects of varying squat depths and movement speeds on dynamic postural stability during the Part the Wild Horse’s Mane (PWHM) movement. Thirteen male participants (age: 25.86 ± 1.35 years; height: 174.26 ± 6.09 cm; body mass: 68.64 ± 8.15 kg) performed the PWHM movement at three different squat heights, high squat (HS), middle squat (MS), low squat (LS), and two different speeds, fast and slow. Dynamic postural stability (DPSI) was assessed through the center-of-mass (CoM) trajectory and the center-of-pressure (CoP) trajectory. The analyses used two-factor repeated-measures ANOVA and statistical nonparametric mapping, with key metrics including anteroposterior stability (APSI), mediolateral stability (MLSI), vertical stability (VSI), DPSI indices, and the path lengths of the CoP and CoM. LS exhibited significantly greater CoP and CoM path lengths compared with MS and HS (*p* < 0.01). Furthermore, fast movements demonstrated higher VSI and DPSI than slow movements (*p* < 0.05). Tai Chi with different squat depths and speeds can affect postural stability. To reduce the fall risk, older adults and individuals with balance impairments should prioritize slower Tai Chi movements, particularly when using high squat postures.

## 1. Introduction

Postural stability refers to the body’s intrinsic ability to maintain, attain, or regain balance through coordinated neuromuscular control [1]. It requires managing the center of mass (CoM) within the base of support (BOS) to maintain equilibrium in both static and dynamic states [2]. Postural stability is crucial for assessing lower-limb musculoskeletal health and evaluating exercise or rehabilitation effectiveness [3]. Impaired postural control can disrupt daily balance and orientation, leading to reduced mobility and heightened fall risk [4]. In addition, studies have found that postural sway is associated with sports injuries in adolescents and adults [4].

In clinical practice, the center of pressure (CoP) is a key marker of balance control, as its dynamic variations reflect postural regulation capacity [5]. CoP fluctuations in the mediolateral (ML) or anteroposterior (AP) directions indicate the degree of balance control, with excessive sway reflecting impaired stability [6,7,8]. The CoM-CoP interaction is also widely accepted as a valid index of dynamic stability during movement [9,10]. Additionally, the dynamic postural stability index (DPSI) is a comprehensive measurement metric that integrates the mediolateral stability index (MLSI), anteroposterior stability index (APSI), and vertical stability index (VSI) [11], and has been demonstrated to possess good reliability [12]. Its magnitude is determined by both the kinematics of the lower-limb joints and the magnitude of the ground reaction force (GRF); a higher DPSI represents worse postural stability [13]. Compared with a single indicator that tends to overlook defects in a specific direction, multidimensional indicators can comprehensively quantify the spatial directionality of dynamic posture stability and increase the reliability of result interpretation.

Exercise plays a vital role in preventing falls. Studies have shown that exercise can effectively prevent falls [14]. In addition, early balance exercises help maintain postural stability and prevent future falls [15]. Meanwhile, as most falls occur during physical activity [16], it is essential to understand postural stability under dynamic exercise conditions. Originating as a Chinese martial art, Tai Chi has evolved into a balance-enhancing mind–body exercise [17,18]. It has garnered widespread attention due to its simplicity, ease of learning, and potential to enhance physical function, thereby reducing disease risk [19,20,21]. Studies have found that Tai Chi can enhance muscle strength, prevent falls, and improve balance [22,23,24]. Song et al. [25] demonstrated significantly reduced mediolateral CoM–CoP separation during dual-task performance in experienced Tai Chi practitioners, correlating with a decreased fall risk. Huang et al. [26] found that long-term Tai Chi practitioners had smaller CoM–CoP separation when crossing obstacles than non-Tai Chi practitioners.

Tai Chi movements at varying squat depths and speeds exhibit distinct biomechanical profiles that may elicit differential neuromuscular adaptations influencing postural stability. Previous studies have shown that slower Tai Chi involves longer activation times for the lower-limb muscles and places higher demands on muscle strength [27]. Wu et al. [28] suggested that, compared with normal walking, Tai Chi gait is characterized by a slower movement speed, greater mediolateral center-of-pressure (CoP) displacement, and a larger lateral shift of the body, thereby resulting in increased postural instability. Our previous study found that different squat heights had differences in hip adduction moment, so different squat heights may have differences in postural stability [29]. However, existing studies have mostly analyzed the biomechanical differences caused by squat depth or speed in isolation and lack a systematic assessment of the interaction between the two on multi-dimensional body stability.

The “Part the Wild Horse’s Mane” (PWHM) is a fundamental component in Tai Chi, characterized by alternating single-stance and double-stance phases. This movement pattern is distinguished by rhythmic anteroposterior displacement of the CoM during lunge transitions. This change in front and back positions leads to a dynamic change in the BOS, which impacts dynamic postural stability [29]. Compared with other movements, PWHM requires greater muscular effort and better reflects functional balance challenges during Tai Chi practice [30]. While prior research has highlighted the biomechanical relevance of either movement speed or squat depth, few studies have explored how these two parameters jointly influence dynamic postural stability in Tai Chi. Moreover, no study to date has employed comprehensive stability indices to quantify their combined effects during functionally representative movements. As long-term Tai Chi training is known to enhance postural stability [17], novice practitioners with limited experience were recruited to ensure a more sensitive assessment of how squat depth and movement speed influence balance performance.

Therefore, this study aimed to explore the effects of different squat depths and different speeds of PWHM on postural stability. We hypothesized that a low squat (LS) and slow speeds would increase postural instability due to greater joint moments. Furthermore, we predicted an interaction between squat depth and speed: slow movements would exacerbate instability at an LS depth due to increased joint loads, while fast movements would increase instability at medium squat (MS) and high squat (HS) depths due to greater inertia.

## 2. Materials and Methods

### 2.1. Study Design

This study was a cross-sectional observational investigation conducted to examine the effects of squat depth and movement speed on dynamic postural stability during Tai Chi. This manuscript was prepared in accordance with the STROBE (Strengthening the Reporting of Observational Studies in Epidemiology) guidelines. This study was approved by the Ethics Committee of Ningbo University (approval no. R20231218), and all participants provided written informed consent before participation.

### 2.2. Participants

Due to the current lack of empirical evidence quantifying the effects of varying squat depths and movement speeds in Tai Chi on DPSI, a post hoc power analysis was conducted based on the observed effect sizes from the collected data, using G * Power 3.1.9.7. For the main effect of squat depth (partial η^2^ = 0.267, equivalent to Cohen’s f = 0.604), the minimum required sample size to achieve 80% power at an alpha level of 0.05 was estimated to be 5 participants. This estimation was based on a two-way repeated measures ANOVA design (3 squat depths × 2 movement speeds), assuming a moderate correlation between repeated measures (α = 0.5) and no violation of sphericity. The actual sample size employed in this study (N = 13) exceeded the minimum requirement derived from the power analysis, thereby ensuring adequate statistical power (1 − β > 0.80) and reinforcing the reliability of the observed effects.

Healthy male novice practitioners (N = 13) were recruited through targeted advertisements distributed via university bulletin boards and social media platforms. Novices were defined as individuals who had practiced Tai Chi for less than six months and fewer than two sessions per week [29]. Participants had a mean age of 25.86 ± 1.35 years, height of 174.26 ± 6.09 cm, body mass of 68.64 ± 8.15 kg, and Tai Chi experience of 4.45 ± 0.61 months. The inclusion criteria were as follows: (1) healthy males aged 20–30 years; (2) novice Tai Chi practitioners, as defined above; (3) no participation in other structured balance training programs within the past six months; and (4) the ability to perform the PWHM movement without pain or functional limitation. The exclusion criteria included the following: (1) a history of musculoskeletal injury, surgery, or disorders affecting the lower extremities within the past six months; (2) any diagnosed neurological, vestibular, or balance-related disorders; and (3) prior professional training in martial arts or dance that may influence postural control.

### 2.3. Experimental Procedures

**Figure 1:**
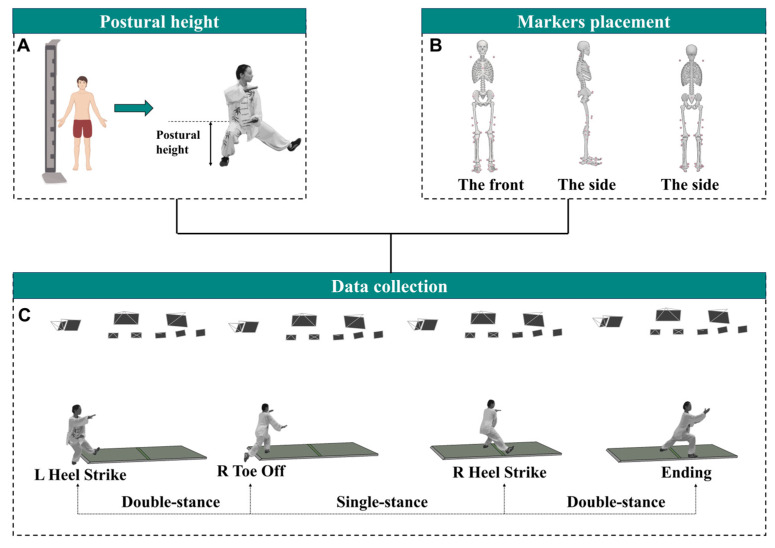
Main test process. (**A**) Determine different squat depths for different participants based on their height; (**B**) placement of the retroreflective markers; (**C**) PWHM dynamic data collection.

All participants completed a single laboratory visit lasting approximately 90 min. Upon arrival, participants underwent eligibility screening to confirm that they met the inclusion and exclusion criteria, followed by anthropometric measurements, including height and body mass. Next, a familiarization session was conducted, during which participants practiced the designated squat depths and movement speeds to ensure an understanding of the experimental protocol. Afterward, a standardized 10 min warm-up was completed, comprising 6 min of PWHM movements and 4 min of static stretching. The squat depths were normalized to individual heights, with HS, MS, and LS defined as 0.81, 0.89, and 0.97 times the standing height, respectively (Figure 1A) [29,31]. A total of 38 retroreflective markers (14 mm in diameter) were then placed on specific anatomical landmarks according to the OpenSim Gait 2392 model (Figure 1B) [32,33]. During the experiment, participants performed PWHM movements under 6 randomized conditions (3 squat depths × 2 movement speeds), with three successful repetitions collected per condition. All trials were performed barefoot and in tight-fitting clothing.

Kinematic data were collected at 200 Hz using a ten-camera motion capture system (Oxford Metrics Ltd., Oxford, UK), while kinetic data were recorded at 1000 Hz using two AMTI force platforms (Advanced Mechanical Technology, Inc., Watertown, MA, USA) [34,35,36]. Movement speed was standardized by synchronizing participants’ performance with auditory cues derived from Tai Chi music. Specifically, the music segment used corresponded to the PWHM movement from the standardized 24-form Tai Chi routine. In the slow-speed condition, participants were instructed to perform the PWHM movement in synchrony with the extended version of the audio segment, whose duration was adjusted to be 1.2 times longer than that of the fast-speed condition [37].

The PWHM movement cycle was divided into five distinct phases based on foot contact patterns: left heel strike (LHS), right toe off (RTO), right heel strike (RHS), left toe off (LTO), and subsequent LHS [27,38]. Given the movement’s bilateral symmetry, analyses focused on the left side and included three key phases: LHS, RTO, and RHS (Figure 1C). Participants positioned each foot on separate force platforms, with data acquisition initiated when the vertical ground reaction force (vGRF) exceeded 10 N during LHS [34]. To preserve natural movement characteristics, participants were instructed to perform continuous cycles without pausing between transitions [39].

### 2.4. Data Processing

In order to analyze postural stability, the following parameters were calculated: (1) stability indices, including ML, AP, and vertical stability indices (MLSI, APSI, VSI, and DPSI); (2) CoP kinematics, comprising the CoP path length, the sway distance along the x-axis (CoP D-x) and y-axis (CoP D-y), and the 95% confidence ellipse area of CoP movements (95% area); (3) CoM kinematics, including the CoM path length and sway distances along the x-axis (CoM D-x), y-axis (CoM D-y), and z-axis (CoM D-z); and (4) CoM-CoP separation parameters, namely, the anteroposterior CoM-CoP separation and its peak value, as well as the mediolateral CoM-CoP separation and its peak value.

Marker trajectories were processed using Vicon Nexus software (version 2.1, Vicon, Oxford, UK) and subsequently analyzed in Visual3D (v.6, C-Motion, Germantown, MD). A fourth-order, zero-lag Butterworth filter was applied to the data, with cutoff frequencies set at 8 Hz for kinematic and 50 Hz for kinetic measurements [40]. GRFs were normalized by body weight, while the CoM and CoP trajectories were normalized by body height. All time-series data underwent temporal normalization to 101 data points across the movement cycle [41]. Postural stability metrics were quantified by analyzing the normalized GRF, CoM, and CoP parameters. The DPSI is a composite measure that combines the MLSI, the APSI, and the VSI. A higher DPSI value indicates poorer postural stability [12,13]. Furthermore, postural control dynamics were quantified through the integrative kinematic analysis of CoM and CoP trajectories. The following equations define the biomechanical relationships:

(1) $$MLSI=\sqrt{\frac{\sum (0-y{)}^{2}}{\mathrm{number}\;\mathrm{of}\;\mathrm{data}\;\mathrm{points}}}\;$$

(2) $$APSI=\sqrt{\frac{\sum (0-x{)}^{2}}{\mathrm{number}\;\mathrm{of}\;\mathrm{data}\;\mathrm{points}}}\;$$

(3) $$VSI=\sqrt{\frac{\sum (0-z{)}^{2}}{\mathrm{number}\;\mathrm{of}\;\mathrm{data}\;\mathrm{points}}}$$

(4) $$DPSI=\sqrt{\frac{\sum (0-GRFx{)}^{2}+\sum (0-GRFy{)}^{2}+\sum (0-GRFz{)}^{2}}{\mathrm{number}\;\mathrm{of}\;\mathrm{data}\;\mathrm{points}}}$$

In these equations, the *x*-, *y*-, and *z*-axes were defined as the ML, AP, and vertical dimensions of the GRF. The denominator for normalization comprised the total frames recorded during the standing phase.

Based on the time series data of the CoP, the swing distance of the CoP in the AP and ML directions, the CoP path length, and 95% area were calculated. The smaller these values, the more stable the posture [42].

### 2.5. Statistical Analysis

Statistical analyses were performed using SPSS 26.0 (IBM Corp., Armonk, NY, USA), R 4.3.1 (R Foundation for Statistical Computing, Vienna, Austria), and MATLAB R2023a (MathWorks Inc., Natick, MA, USA) with the SPM1D toolbox. All data are presented as means ± standard deviations (SDs). A significance threshold of *p* < 0.05 was applied to all statistical tests.

The normality of the data was assessed using the Shapiro–Wilk test, and the homogeneity of variances was evaluated using Levene’s test. For variables that met parametric assumptions, a two-factor repeated-measures analysis of variance (ANOVA) was conducted to examine the main and interaction effects of the squat depth (HS, MS, or LS) and movement speed (fast or slow) on all dependent variables. Sphericity was tested using Mauchly’s test, and when it was violated, Greenhouse–Geisser corrections were applied. Significant interactions were followed by Bonferroni-adjusted post hoc tests for pairwise comparisons both within and between conditions. In the absence of significant interactions, main effects were interpreted independently. Effect sizes were estimated using η^2^, with values of 0.01 ≤ η^2^ < 0.06 indicating a small effect, 0.06 ≤ η^2^ < 0.14 indicating a medium effect, and η^2^ ≥ 0.14 indicating a large effect [43]. For variables that violated the assumption of normality, nonparametric analyses were employed. Specifically, the aligned rank transform (ART) method was used to perform a nonparametric two-way repeated-measures ANOVA while retaining the ability to interpret interactions and main effects. ART analyses were conducted using the ARTool package in R, and post hoc comparisons were Bonferroni-corrected.

The relationship between DPSI and APSI, MLSI, and VSI was compared using linear regression analysis. Additionally, statistical nonparametric mapping (SnPM) was used to perform a nonparametric two-factor repeated-measures ANOVA.

## 3. Results

Since several outcome measures—including APSI, MLSI, VSI, DPSI, the CoM displacement in the x-, y-, and z-directions (CoM D-x, D-y, and D-z), the AP and ML CoM-CoP separations, and their peak values—violated the assumption of normality, the aligned rank transform (ART) method was used to evaluate the main and interaction effects within a non-parametric factorial design.

### 3.1. Dynamic Postural Steadiness Index

The statistical analyses showed significant effects of the squat depth and movement speed on the DPSI parameters (Table 1). For APSI, there was no significant speed × depth interaction (*p* > 0.05). There was a significant main effect of squat depth (*p* < 0.01). Post hoc comparisons showed that the HS was significantly lower than the MS and LS (*p* < 0.01). The MS was significantly lower than the LS (*p* < 0.01).

For MLSI, there was no significant speed × depth interaction (*p* > 0.05). There was a significant main effect of squat depth (*p* < 0.01). Post hoc comparisons showed that the HS was significantly lower than the LS (*p* < 0.01). The MS was significantly lower than the LS (*p* < 0.01).

For VSI, there was no significant speed × depth interaction (*p* > 0.05). There was a significant main effect of the squat depth and movement speed (*p* < 0.01). Post hoc comparisons revealed that the HS had a significantly lower VSI than the MS (*p* < 0.01). A fast speed had a significantly greater VSI than a slow speed (*p* < 0.05).

For DPSI, there was no significant speed × depth interaction (*p* > 0.05). There was a significant main effect of the squat depth and movement speed (*p* < 0.01). Post hoc comparisons revealed that the HS had a significantly lower DPSI than both the MS and LS (*p* < 0.01). A fast speed had a significantly greater DPSI than a slow speed (*p* < 0.05).

**Figure 2:**
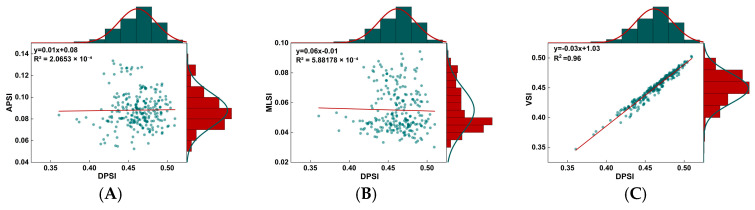
Linear regression analysis of DPSI with APSI, MLSI, and VSI. (**A**) Linear correlation analysis between DPSI and APSI; (**B**) linear correlation analysis between DPSI and MLSI; (**C**) linear correlation analysis between DPSI and VSI.

The relationship between DPSI and APSI, MLSI, and VSI was evaluated using linear regression analysis (Figure 2). The results showed a significant correlation between DPSI and VSI (*p* < 0.001; R^2^ = 0.96).

### 3.2. Center of Pressure

Statistical analyses revealed significant effects of squat depth and movement speed on CoP parameters (Table 2). For the CoP path length, there was no significant speed × squat depth interaction (*p* > 0.05). There were significant main effects of squat depth and movement speed (*p* < 0.01). Post hoc comparisons revealed that the HS had significantly lower path lengths than both the MS and LS (*p* < 0.01). The MS had significantly lower path lengths than the LS (*p* < 0.01). A fast movement speed resulted in significantly lower path lengths than a slow speed (*p* < 0.01).

For the sway distance of the CoP in the x-axis direction (CoP D-x), there was a significant speed × depth interaction (*p* < 0.05). Post hoc comparisons revealed that a fast speed with LS had a significantly greater CoP D-x than both the HS and MS (*p* < 0.01). Under HS conditions, a slow speed resulted in a significantly greater CoP D-x than a fast speed (*p* < 0.05).

For the sway distance of the CoP in the y-axis direction (CoP D-y), there was no significant speed × depth interaction (*p* > 0.05). There was a significant main effect of the squat depth (*p* < 0.001). The LS had a significantly greater CoP D-y than both the MS and HS (*p* < 0.01).

For the 95% confidence ellipse area of the CoP movements (95% area), there was no significant speed × depth interaction (*p* > 0.05). There was a significant main effect of the squat depth (*p* < 0.01). The HS had a significantly lower 95% area than both the MS and LS (*p* < 0.01). The MS had a significantly lower 95% area than the LS (*p* < 0.01).

**Figure 3:**
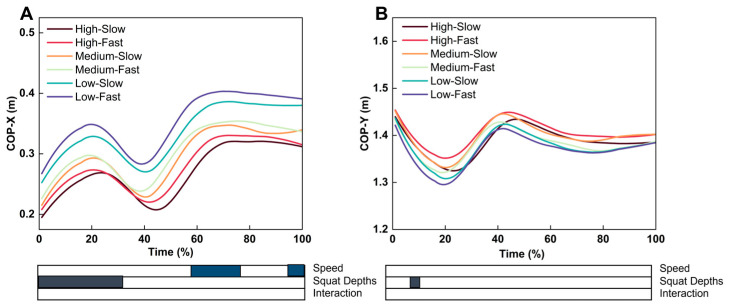
Effects of squat speed and depth on CoP-X and CoP-Y trajectories. (**A**) Trajectory changes in CoP-X under different speed and depth combinations; (**B**) trajectory changes in CoP-Y under different speed and depth combinations.

For the CoP position on the X-axis, the SnPM analysis showed significant differences in movement speed during the 58–75% and 96–100% ranges of the standing phase. There was a significant difference in the squat depth during 0–30% of the standing phase (Figure 3). For the CoP position on the Y-axis, the SnPM analysis showed a significant difference in the squat depth during the 11–13% range of the standing phase (Figure 3).

### 3.3. Center of Mass

The statistical analyses showed significant effects of the squat depth and movement speed on the CoM parameters (Table 3). For the CoM path length, there was no significant speed × squat depth interaction (*p* > 0.05). There was a significant main effect of the squat depth and movement speed (*p* < 0.01). Post hoc comparisons revealed that the LS had significantly greater CoM path lengths than both the MS and HS (*p* < 0.01). The MS had significantly greater CoM path lengths than the HS (*p* < 0.01). A slow speed had significantly greater CoM path lengths than a fast speed (*p* < 0.01).

For the sway distance of the CoM in the x-axis direction (CoM D-x), there was no significant speed × squat depth interaction (*p* > 0.05). There was a significant main effect of the squat depth and movement speed (*p* < 0.01). Post hoc comparisons revealed that the LS had a significantly greater CoM D-x than both the MS and HS (*p* < 0.01). The MS had a significantly greater CoM D-x than the HS (*p* < 0.01). A slow speed had a significantly greater CoM D-x than a fast speed (*p* < 0.01).

For the sway distance of the CoM in the y-axis direction (CoM D-y), there was no significant speed × squat depth interaction (*p* > 0.05). There was a significant main effect of the squat depth (*p* < 0.01). Post hoc comparisons revealed that the LS had a significantly greater CoM D-y than both the MS and HS (*p* < 0.01). The MS had a significantly greater CoM D-y than the HS (p < 0.01).

For the sway distance of the CoM in the z-axis direction (CoM D-z), there was no significant speed × squat depth interaction (*p* > 0.05). There was a significant main effect of the squat depth and movement speed (*p* < 0.01). Post hoc comparisons revealed that the LS had a significantly greater CoM D-z than both the MS and HS (*p* < 0.01). The MS had a significantly greater CoM D-z than the HS (*p* < 0.01). A slow speed had a significantly greater CoM D-z than a fast speed (*p* < 0.01).

**Figure 4:**
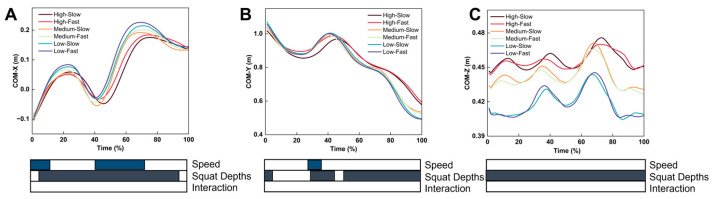
Effects of squat speed and depth on CoM-X, CoM-Y, and CoM-Z trajectories. (**A**) The trajectory changes in CoM-X under different speed and depth combinations; (**B**) the trajectory changes in CoM-Y under different speed and depth combinations; (**C**) the trajectory changes in CoM-Z under different speed and depth combinations.

For the CoM position on the X-axis, the SnPM analysis showed significant differences in the movement speed during the 0–11% and 42–70% ranges of the standing phase. There was a significant difference in the squat depth during the 6–95% range of the standing phase (Figure 4). For the CoM position on the Y-axis, the SnPM analysis showed significant differences in the movement speed during the 28–36% range of the standing phase. There was a significant difference in the squat depth during the 0–3%, 30–41%, and 48–100% ranges of the standing phase (Figure 4). For the CoM position on the Z-axis, the SnPM analysis showed significant differences in the squat depth during the 0–100% range of the standing phase (Figure 4).

### 3.4. CoM-CoP Separation

The statistical analyses showed significant effects of the squat depth and movement speed on the CoM-CoP separation parameters (Table 4). For AP CoM-CoP separation, there was no significant speed × squat depth interaction (*p* > 0.05). There was a significant main effect of squat depth (*p* < 0.01). Post hoc comparisons revealed that MS had significantly greater AP CoM-CoP separation than the HS (*p* < 0.05).

For the peak AP CoM-CoP separation, there was no significant speed × squat depth interaction (*p* > 0.05). There was a significant main effect of the squat depth (*p* < 0.01). Post hoc comparisons revealed that the LS had a significantly greater peak AP CoM-CoP separation than both the MS and HS (*p* < 0.01). The MS had a significantly greater peak AP CoM-CoP separation than the HS (*p* < 0.01).

For the ML CoM-CoP separation, there was no significant speed × squat depth interaction (*p* > 0.05). There was a significant main effect of the squat depth (*p* < 0.01). Post hoc comparisons revealed that the LS had a significantly greater ML CoM-CoP separation than both the MS and HS (*p* < 0.01). The MS had a significantly greater ML CoM-CoP separation than the HS (*p* < 0.01).

For the peak ML CoM-CoP separation, there was no significant speed × squat depth interaction (*p* > 0.05). There was a significant main effect of the squat depth (*p* < 0.01). Post hoc comparisons revealed that the LS had a significantly greater peak ML CoM-CoP separation than both the MS and HS (*p* < 0.01). The MS had a significantly greater peak ML CoM-CoP separation than the HS (*p* < 0.01).

## 4. Discussion

This study investigated the effects of squat depth and movement speed in Tai Chi on dynamic postural stability. Lower squat depths, particularly an LS, significantly increased instability, as indicated by the elevated APSI, MLSI, VSI, DPSI, CoP path length, 95% area, and CoM displacements. Fast movements resulted in a higher VSI, DPSI, and CoP path length, reflecting impaired dynamic stability. In contrast, slow movements led to larger CoM excursions, indicating greater postural demands and continuous compensatory adjustments. A significant interaction was found only for CoP displacement in the mediolateral direction. These findings suggest that the squat depth and movement speed impose distinct and direction-specific demands on postural control, with slow movements requiring sustained regulatory strategies to maintain balance.

Dynamic stability is more closely related to falls than static movement stability, and most falls occur under dynamic conditions [44]. PWHM is a typical dynamic movement highly represented in Tai Chi. It consists of a single-support period and a double-support period, with the single-support period having a higher proportion (Figure 1C). This study showed that different squat heights had different effects on postural stability. Overall, the LS was more demanding on postural stability than the MS and HS. For example, consistent with our research hypothesis, our study found that the LS had greater APSI and MLSI, as well as a greater CoP path length and CoM path length, compared with the HS. This suggests that an LS leads to greater postural instability, and the body needs to make more frequent adjustments to maintain postural stability. In addition, the LS had a greater peak AP CoM-CoP separation, ML CoM-CoP separation, and peak ML CoM-CoP separation than the MS and HS. Previous studies have shown that enhanced postural stability is achieved when the CoM is closer to the CoP, whereas an excessive separation between the CoM and CoP is associated with a heightened risk of falls. Therefore, compared with the MS and HS, the LS imposes higher demands on postural stability [45]. Excessive loads on lower-limb joints may lead to compensatory movement adjustments in the trunk and lower-limb joints [46], resulting in greater postural sway [47,48], which is consistent with our study. Our previous studies have demonstrated that as the depth of squatting increases, the load on the joints becomes progressively greater, as well as the demand for lower-limb muscle strength [29]. The quadriceps and hamstrings play an important role in supporting body weight, and insufficient quadricep and hamstring strength significantly increases the risk of falls [49]. Therefore, compared with the MS and HS, the LS increases joint loading, demands greater lower-limb muscle strength, and, consequently, impairs postural stability. Furthermore, contrary to our hypothesis, we found that the MS had higher VSI and DPSI than the LS, although there were no significant differences. Considering that DPSI is a composite variable and is sensitive to changes in all directions [50], we analyzed the relationship between MLSI, APSI, VSI, and DPSI in this study. The results showed a significant correlation between VSI and DPSI, indicating that the dynamic stability between different squat heights was mainly attributed to stability in the vertical direction. The biomechanical factors that have the greatest impact on balance are the size and quality of the support surface [1]. Therefore, we believe that, compared with the MS, the LS provides a larger BOS, and the projection of the body’s CoM is closer to the boundary of the foot support area, thereby conferring superior postural stability.

Different movement speeds also affected postural stability. The CoP path length is reliable in assessing postural stability [51]. Our study found that compared with fast Tai Chi, slow Tai Chi had a larger CoP path length and CoM path length. The SPM results showed that the differences in the positions of the CoP and CoM at different movement speeds mainly occurred in the single support phase of PWHM and were predominantly manifested in the coronal plane. The variation in the exercise speed affects the load on the joints and may also alter muscle activation patterns [27]. Wu et al. [27] found that compared with faster Tai Chi, slower Tai Chi involves longer activation times for the lower-limb muscles and places higher demands on muscle strength. Therefore, a possible explanation is that slow PWHM increases the single-stance phase time, leading to increased joint loading and, thus, reduced postural stability in the frontal plane. Previous studies have found that higher lateral impulses impair coronal plane stability, increasing joint wear and elevating the fall risk [52,53]. Therefore, slower speeds of PWHM have higher demands on postural stability than faster speeds. Meanwhile, fast Tai Chi exhibited higher VSI and DPSI values compared with slow Tai Chi, indicating reduced dynamic stability. Faster movements generate greater acceleration and momentum, leading to increased inertial forces. Consistent with our findings, previous gait studies have shown that the peak GRF rises significantly with increased walking speed [54], while slower speeds are associated with enhanced stability [55]. Accordingly, we infer that during fast execution of PWHM, the supporting leg must absorb and redirect larger ground reaction forces, resulting in elevated VSI and DPSI.

This study had several limitations. First, while Tai Chi practice is beneficial for adults, falls predominantly occur in middle-aged and older populations; thus, future research should specifically investigate these age groups. Second, the inclusion of novice practitioners may introduce movement variability, which could confound the biomechanical effects of squat depth and speed on postural stability. Additionally, the exclusively male cohort and limited sample size restrict the generalizability of the findings. Future studies should recruit diverse cohorts, encompassing individuals of varying genders, ages, and skill levels, to enhance the external validity and clinical applicability of the results. Finally, as muscle strength and joint loading are critical determinants of postural stability, future studies should quantify their interactions through integrated biomechanical and physiological assessments. Nevertheless, this study provides foundational evidence that Tai Chi squat parameters modulate postural stability, informing strategies to mitigate fall risks.

Based on the above research results, for individuals at high risk of falling, we recommend practicing slow Tai Chi in combination with an HS to reduce the occurrence of falls. For healthy adults, slow Tai Chi with an MS and LS can be performed to improve muscle strength and dynamic posture stability, thereby improving balance ability.

## 5. Conclusions

This study investigated how squat depth and movement speed influence dynamic postural stability during the Tai Chi movement PWHM. We found that an LS and slow speed were associated with longer CoP and CoM path lengths, reflecting more frequent postural adjustments. Fast-speed Tai Chi resulted in higher DPSI and VSI, indicating poorer stability. These findings suggest that slow and deep-squat Tai Chi may be safer for populations with impaired balance, while healthy individuals may benefit from a low squat depth and slow speed to enhance dynamic stability.

## Figures and Tables

**Table 1:** Differences in PWHM between APSI, MLSI, VSI, and DPSI at different squat depths and different movement speeds.

	Squat Depth	Slow	Fast	Squat Depth		Speed		Squat Depth × Speed	
				*p*	η^2^	*p*	η^2^	*p*	η^2^
APSI (BW)	High	0.075 ± 0.013	0.081 ± 0.010	0.001 ^a^	0.442	0.179	0.008	0.106	0.020
	Medium	0.088 ± 0.010	0.088 ± 0.010						
	Low	0.100 ± 0.017	0.100 ± 0.018						
MLSI (BW)	High	0.050 ± 0.010	0.052 ± 0.010	0.001 ^a^	0.114	0.357	0.004	0.409	0.008
	Medium	0.054 ± 0.014	0.053 ± 0.011						
	Low	0.060 ± 0.017	0.061 ± 0.020						
VSI (BW)	High	0.440 ± 0.029	0.442 ± 0.027	0.001 ^a^	0.077	0.024 ^b^	0.023	0.708	0.003
	Medium	0.452 ± 0.023	0.461 ± 0.016						
	Low	0.447 ± 0.025	0.452 ± 0.026						
DPSI (BW)	High	0.449 ± 0.029	0.453 ± 0.026	0.001 ^a^	0.108	0.013 ^b^	0.027	0.764	0.002
	Medium	0.464 ± 0.021	0.473 ± 0.016						
	Low	0.463 ± 0.022	0.468 ± 0.022						

Note: ^a^ indicates a significant difference between different squat depths; ^b^ indicates a significant difference between different movement speeds.

**Table 2:** Differences in PWHM between CoP path length, CoP D-x, CoP D-y, and CoP 95% area at different squat depths and different movement speeds.

	Squat Depth	Slow	Fast	Squat Depth		Speed		Squat Depth × Speed	
				*p*	η^2^	*p*	η^2^	*p*	η^2^
Path length (mm)	High	849.205 ± 112.985	764.129 ± 90.451	0.001 ^a^	0.378	0.001 ^b^	0.160	0.082	0.032
	Medium	905.681 ± 110.559	823.961 ± 93.739						
	Low	944.438 ± 87.953	910.002 ± 105.934						
CoP _D-x_ (mm)	High	152.992 ± 16.090	145.555 ± 9.839	-	-	-	-	0.026 ^c^	0.047
	Medium	155.005 ± 12.084	151.309 ± 16.150						
	Low	158.295 ± 11.434	162.020 ± 14.505						
CoP _D-y_ (mm)	High	136.522 ± 22.458	125.836 ± 25.430	0.001 ^a^	0.113	0.322	0.013	0.055	0.037
	Medium	131.903 ± 19.963	129.912 ± 22.673						
	Low	140.053 ± 19.686	140.985 ± 17.846						
95% area (mm^2^)	High	985.045 ± 264.557	906.513 ± 226.123	0.001 ^a^	0.284	0.402	0.009	0.400	0.012
	Medium	1094.852 ± 292.342	1036.017 ± 317.222						
	Low	1225.087 ± 297.009	1242.360 ± 291.745						

Note: ^a^ indicates a significant difference between different squat depths; ^b^ indicates a significant difference between different movement speeds; ^c^ indicates that there is an interaction between squat height and movement speed. Abbreviations: CoP _D-x_, the sway distance of the CoP in the x-axis direction; CoP _D-y_, the sway distance of the CoP in the y-axis direction; 95% area, the 95% confidence ellipse area of the CoP movements.

**Table 3:** Differences in PWHM between CoM path length, CoM D-x, CoM D-y, and CoM _D-Z_ at different squat depths and different movement speeds.

	Squat Depth	Slow	Fast	Squat Depth		Speed		Squat Depth × Speed	
				*p*	η^2^	*p*	η^2^	*p*	η^2^
Path length (m)	High	1.035 ± 0.080	0.968 ± 0.065	0.001 ^a^	0.846	0.001 ^b^	0.146	0.212	0.041
	Medium	1.121 ± 0.112	1.077 ± 0.063						
	Low	1.220 ± 0.072	1.184 ± 0.065						
CoM _D-x_ (m)	High	0.286 ± 0.043	0.275 ± 0.043	0.001 ^a^	0.289	0.003 ^b^	0.039	0.202	0.014
	Medium	0.303 ± 0.053	0.292 ± 0.044						
	Low	0.321 ± 0.058	0.324 ± 0.062						
CoM _D-y_ (m)	High	0.448 ± 0.053	0.452 ± 0.041	0.001 ^a^	0.772	0.354	0.004	0.342	0.010
	Medium	0.512 ± 0.048	0.514 ± 0.048						
	Low	0.580 ± 0.034	0.568 ± 0.039						
CoM _D-z_ (m)	High	0.058 ± 0.013	0.054 ± 0.015	0.001 ^a^	0.248	0.001 ^b^	0.045	0.773	0.002
	Medium	0.070 ± 0.019	0.063 ± 0.020						
	Low	0.076 ± 0.017	0.070 ± 0.017						

Note: ^a^ indicates a significant difference between different squat depths; ^b^ indicates a significant difference between different movement speeds. Abbreviations: CoM _D-x_, the sway distance of the CoM in the x-axis direction; CoM _D-y_, the sway distance of the CoM in the y-axis direction; CoM _D-z_, the sway distance of the CoM in the z-axis direction.

**Table 4:** Differences in PWHM between AP CoM-CoP separation, peak AP CoM-CoP separation, ML CoM-CoP separation, and peak ML CoM-CoP separation at different squat depths and different movement speeds.

	Squat Depth	Slow	Fast	Squat Depth		Speed		Squat Depth × Speed	
				*p*	η^2^	*p*	η^2^	*p*	η^2^
AP CoM-CoP separation (m)	High	−0.543 ± 0.073	−0.543 ± 0.050	0.032 ^a^	0.030	0.955	0.001	0.458	0.007
	Medium	−0.567 ± 0.072	−0.556 ± 0.076						
	Low	−0.547 ± 0.054	−0.548 ± 0.062						
Peak AP CoM-CoP separation (m)	High	0.820 ± 0.102	0.821 ± 0.072	0.001 ^a^	0.298	0.524	0.002	0.502	0.006
	Medium	0.874 ± 0.093	0.867 ± 0.085						
	Low	0.897 ± 0.070	0.896 ± 0.068						
ML CoM-CoP separation (m)	High	−0.203 ± 0.029	−0.204 ± 0.027	0.001 ^a^	0.329	0.399	0.003	0.209	0.014
	Medium	−0.220 ± 0.039	−0.217 ± 0.049						
	Low	−0.241 ± 0.044	−0.251 ± 0.060						
Peak ML CoM-CoP separation (m)	High	0.301 ± 0.048	0.300 ± 0.043	0.001 ^a^	0.298	0.872	0.001	0.266	0.012
	Medium	0.323 ± 0.061	0.326 ± 0.061						
	Low	0.353 ± 0.071	0.364 ± 0.086						

Note: ^a^ indicates a significant difference between different squat depths. Abbreviations: AP, anterior–posterior; ML, mediolateral.

## Data Availability

The original contributions presented in this study are included in this article. Further inquiries can be directed to the corresponding authors.

## Abbreviations

The following abbreviations were used in this manuscript:

PWHM	Part the Wild Horse’s Mane
HS	High squat
MS	Middle squat
LS	Low squat
CoM	Center of mass
CoP	Center of pressure
GRF	Ground reaction forces
DPSI	Dynamic postural stability index
MLSI	Mediolateral stability index
APSI	Anteroposterior stability index
VSI	Vertical stability index
BOS	Base of support
ML	Mediolateral
AP	Anteroposterior
LHS	Left heel strike
RTO	Right toe off
RHS	Right heel strike
LTO	Left toe off
SnPM	Statistical nonparametric mapping
COG	Center of gravity
